# Efficacy and health economics of Bufei Yishen granules in patients with frequent exacerbator phenotype in the stable phase of chronic obstructive pulmonary disease: study protocol for a multicenter, randomized, double-blind, placebo-controlled trial

**DOI:** 10.3389/fmed.2025.1662655

**Published:** 2025-09-03

**Authors:** Qionghua Xiao, Minghang Wang, Zegeng Li, Tao Chen, Jiansheng Li

**Affiliations:** ^1^National Regional TCM (Pulmonary Disease) Diagnostic and Treatment Center, The First Affiliated Hospital of Henan University of Chinese Medicine, Zhengzhou, China; ^2^The First Clinical Medical School, Henan University of Chinese Medicine, Zhengzhou, China; ^3^Anhui University of Chinese Medicine, Hefei, China; ^4^Liverpool School of Tropical Medicine, Liverpool, United Kingdom; ^5^Henan University of Chinese Medicine, Zhengzhou, China

**Keywords:** stable phase of chronic obstructive pulmonary disease, frequent exacerbator phenotype, Bufei Yishen granules, randomized controlled trial, study protocol

## Abstract

**Introduction:**

Patients with frequent exacerbator phenotype in chronic obstructive pulmonary disease (COPD) tend to experience a progressive decline in lung function, a gradual deterioration of the disease, and even a serious threat to their lives. However, current treatment measures still need further improvements to reduce the frequency of acute exacerbation of chronic obstructive pulmonary disease (AECOPD). Therefore, our team developed Bufei Yishen (BFYS) granules specifically for patients with frequent exacerbator phenotype in COPD and is conducting a randomized controlled trial (RCT) to validate their effectiveness.

**Methods:**

A multi-center, randomized, double-blind, placebo-controlled trial will be conducted. A total of 848 patients will participate in the study, with a treatment duration of 1 year. The participants will be randomly assigned to the experimental group and the control group in a 1:1 ratio. Both groups will receive health education and conventional drugs. In addition, the experimental group will receive BFYS granules, while the control group will be given the corresponding BFYS placebo. The primary outcome is the frequency of AECOPD. The secondary outcomes include the frequency of AECOPD leading to hospitalization, the mortality rate, lung function, six-minute walk distance (6MWD), clinical symptoms and signs scores, and quality of life. Safety outcomes include vital signs and laboratory tests. Statistical analysis will be conducted using SPSS software (version 25.0). Furthermore, the health economics evaluation of the BFYS granules will use cost-effectiveness analysis methods.

**Ethics and dissemination:**

The study protocol was approved by the Ethics Committee of the First Affiliated Hospital of Henan University of Chinese Medicine (No. 2024HL-043-01). Written informed consent will be obtained from all participants. The results will be published in a peer-reviewed journal after the end of the study. The data of this trial will be disseminated publicly through conferences and publications.

**Clinical trial registration:**

https://clinicaltrials.gov/, identifier NCT06326658.

## 1 Introduction

Chronic obstructive pulmonary disease (COPD) is a common chronic respiratory disease characterized by persistent airflow restriction and respiratory symptoms (1). It has high global incidence and mortality rates (2–4). Epidemiological studies have shown that the prevalence of COPD among individuals aged over 40 years in China is 13.7% (2). It seriously impairs patients’ work capacity and quality of life (5–7). Notably, recent data indicate that COPD became the third leading cause of death worldwide in 2019, leading to approximately 3.23 million deaths annually and imposing a substantial societal burden (8).

The frequent exacerbator phenotype in COPD is defined as experiencing two or more acute exacerbations within the past year (9). Specifically, patients with frequent AECOPD exhibit significantly diminished quality of life, impaired work capacity, worsened clinical symptoms, and declined lung function (10). Currently, conventional therapeutic approaches primarily rely on anti-inflammatory and symptomatic treatments, which require further optimization to effectively reduce AECOPD frequency (11).

In traditional Chinese medicine (TCM), COPD is referred to as “chuan zheng” (dyspnea) or “fei zhang” (lung distension). Its pathogenesis involves deficiency of lung, spleen and kidney qi, accompanied by phlegm and blood stasis blocking the airways. AECOPD often arises from external pathogens, which worsen this imbalance of qi deficiency and phlegm/stasis, exacerbating symptoms (12). TCM exhibits therapeutic potential for COPD (13–15). Previous study has found that lung-kidney qi deficiency is significantly correlated with the frequent exacerbator phenotype in COPD (16). The lung manages qi and respiration; the kidney governs qi reception, so their qi deficiency weakens respiratory function. Phlegm and blood stasis are key pathological products that block airways and worsen symptoms. Drawing on extensive clinical experience, we developed Bufei Yishen (BFYS) granules, which are designed to tonify the lung and kidney, resolve phlegm, and promote blood circulation. Tonifying the lung and kidney enhances respiration by nourishing their qi; resolving phlegm and promoting blood circulation relieves airway obstruction by dissolving phlegm and unblocking blood stasis, thus improving the condition. Our prior clinical investigations have shown that BFYS granules can effectively alleviate clinical symptoms, improve lung function, enhance exercise capacity, and boost patients’ quality of life (17, 18). Concurrently, animal studies have revealed that BFYS granules significantly improve lung function, reduce lung tissue pathological damage, and suppress inflammatory responses in COPD rat models (19–22). Notably, no relevant between-group differences in adverse events were observed in clinical studies, supporting its safety profile (17, 18).

Previous studies have not clarified the impact of BFYS granules on AECOPD frequency and health economic outcomes. To address this evidence gap and generate high-level evidence, we will conduct a multi-center, randomized, double-blind, placebo-controlled trial enrolling COPD patients with the frequent exacerbator phenotype.

## 2 Methods and analysis

### 2.1 Objective

The primary objective of this study is to evaluate the efficacy of BFYS granules in reducing the frequency of AECOPD among stable COPD patients with the frequent exacerbator phenotype. Secondary objectives include evaluating the impact of BFYS granules on key clinical outcomes such as the frequency of AECOPD requiring hospitalization, mortality rates, lung function tests, exercise capacity, clinical symptoms and signs scores, quality of life, and health economic analysis. It is expected that BFYS granules will not only reduce the frequency of AECOPD but also improve lung function, exercise capacity, clinical symptoms, quality of life, and demonstrate favorable cost-effectiveness compared to placebo.

### 2.2 Study design and setting

**Figure 1:**
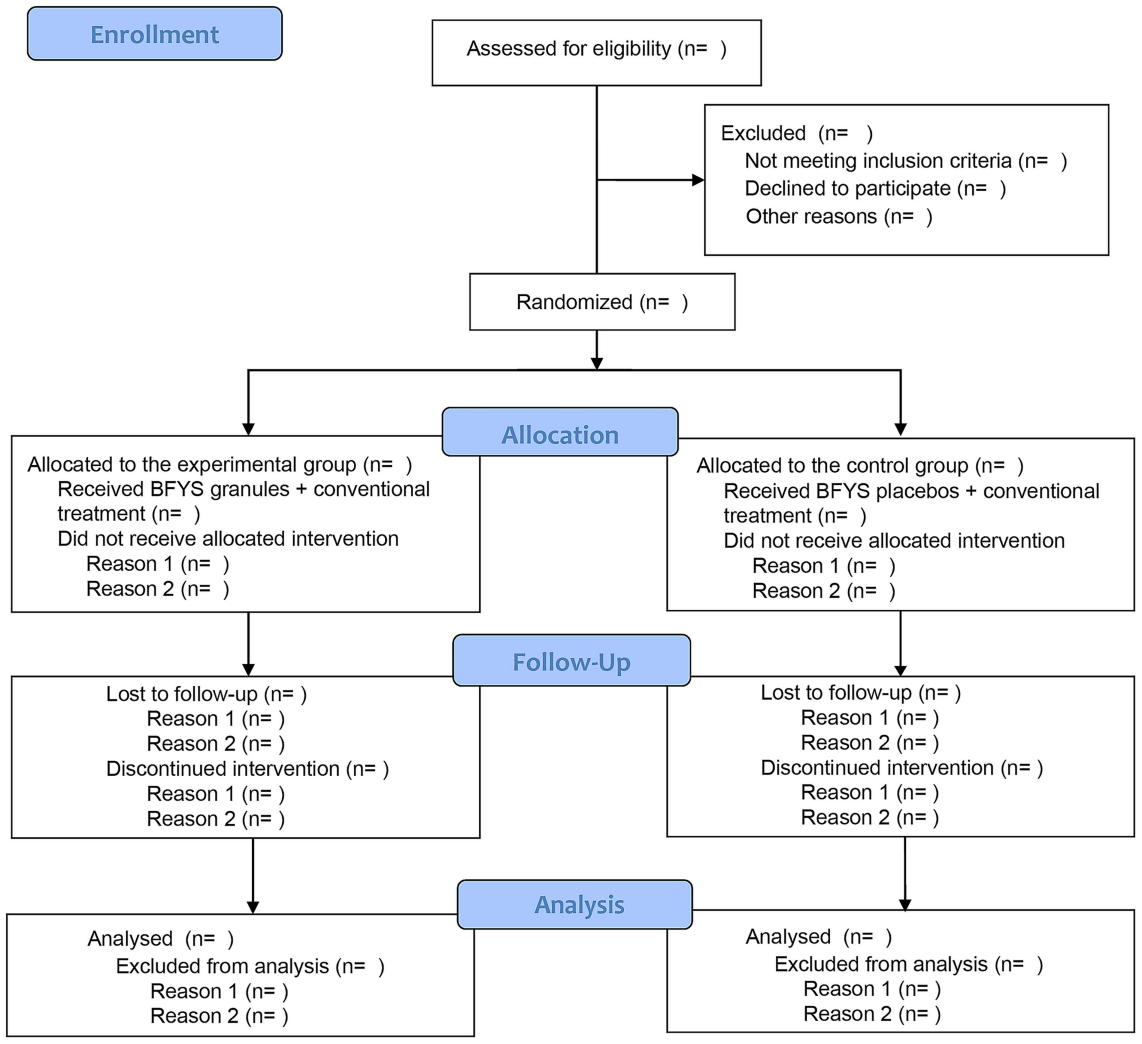
The flow chart for this trial.

This study will conduct a multi-center, randomized, double-blind, placebo-controlled clinical trial. A total of 848 patients with frequent exacerbator phenotype in the stable phase of COPD will participate in this study. After signing the informed consent form, they will be randomly divided into experimental group and control group at a 1:1 ratio. Then, all subjects will receive BFYS granules or placebos for a 52-week intervention period. The flow chart of this trial is demonstrated in Figure 1.

This trial was registered on ClinicalTrials.gov (No. NCT06326658) and adhered to the Standard Protocol Items: Recommendations for Interventional Trials (SPIRIT) 2013 Statement (23). Additional details are available in Supplementary file 1.

### 2.3 Participants

Patients will be recruited from departments of respiratory disease in 13 hospitals, including the First Affiliated Hospital of Henan University of Chinese Medicine, the First Affiliated Hospital of Anhui University of Chinese Medicine, Longhua Hospital affiliated to Shanghai University of Traditional Chinese Medicine, Affiliated Hospital of Liaoning University of Traditional Chinese Medicine, the Second Affiliated Hospital of Liaoning University of Chinese Medicine, the First Affiliated Hospital of Heilongjiang University of Chinese Medicine, Shaanxi Hospital of Traditional Chinese Medicine, Kunming Hospital of Chinese Medicine, Jiangyin Hospital of Traditional Chinese Medicine, Xiangyang Hospital of Traditional Chinese Medicine, Qingdao Hospital of Chinese Medicine, Henan Provincial Hospital of Traditional Chinese Medicine, and Zhuhai Hospital of Integrated Chinese and Western Medicine.

#### 2.3.1 Diagnostic criteria

The diagnostic criteria for the disease are based on the “Global Strategy for Chronic Obstructive Lung Disease (GOLD) 2023” (24) and the “Expert consensus on identification and management of patients at high risk for acute exacerbation of chronic obstructive pulmonary disease in China” (25).

The diagnostic criteria for TCM syndromes are based on the “Diagnostic Criteria for TCM Syndromes of Chronic Obstructive Pulmonary Disease (2011 Edition)” issued by the Chinese Association of Chinese Medicine (26).

#### 2.3.2 Inclusion criteria

Patients enrolled in this trial are required to meet the following criteria:

(1) Diagnosed with COPD in the stable phase.
(2) With the frequent exacerbator phenotype of COPD.
(3) Aged between 40 and 80 years.
(4) With a stable disease condition for at least 4 weeks before enrollment.
(5) Not having participated in other clinical trials within 1 month before enrollment.
(6) Treated with dual bronchodilator or triple inhalation therapy for at least 3 months before enrollment.
(7) Having undergone a 2-week washout period before enrollment.
(8) Voluntarily participating in this trial and signing the informed consent form.

#### 2.3.3 Exclusion criteria

Patients who meet any of the following criteria will be excluded:

(1) Patients with other respiratory diseases, including asthma, active tuberculosis, bronchiectasis, pulmonary hypertension, pulmonary embolism, pneumothorax, pleural effusion, interstitial lung disease, or other intervention-requiring pulmonary conditions.
(2) Patients with severe or acute cardiovascular and cerebrovascular disorders, including acute cardiovascular and cerebrovascular events, malignant arrhythmias, unstable angina, and uncontrolled hypertension.
(3) Patients with severe hepatic or renal diseases (severe liver disease defined as cirrhosis, portal hypertension with variceal bleeding; severe kidney disease requiring dialysis or kidney transplantation).
(4) Patients with abnormal liver or renal function (ALT or AST exceed 1.5 times the upper limit of normal, or Scr exceeds the upper limit of normal).
(5) Patients with a history of tumor surgery, radiotherapy, or chemotherapy within the past 5 years.
(6) Patients with mobility impairment due to severe osteoarthropathy, neuromuscular disorders, or peripheral vascular disease.
(7) Patients with contraindications to 6-min walking test (6MWT), including resting heart rate > 120 bpm, systolic blood pressure > 180 mmHg, or diastolic blood pressure > 100 mmHg.
(8) Patients with cognitive or psychiatric disorders.
(9) Patients who received oral glucocorticoids within 2 weeks prior to enrollment.
(10) Participants who took prohibited Chinese herbal preparations within 2 weeks prior to enrollment.
(11) Patients with known allergies to the study drugs.
(12) Pregnant or lactating women, as well as individuals unable to use effective contraception.

#### 2.3.4 Withdrawal/termination criteria

Patients with any of the following conditions will be withdrawn or terminated from the intervention, but will continue to be followed for outcome assessment and included in the Intention-To-Treat (ITT) analysis according to their original group assignment, unless truly lost to follow-up:

(1) Patients who experience serious adverse reactions or allergies after taking BFYS granules. In this study, serious adverse reactions include events that result in death, are life-threatening, require hospitalization or prolongation of existing hospitalization, or lead to persistent or significant disability/incapacity.
(2) Patients who have disease progression, such as uncontrolled AECOPD or concurrent severe illnesses (e.g., acute cardiovascular events).
(3) Patients who commit serious protocol violations, including unauthorized use of prohibited medications, or ≥4 consecutive weeks of non-adherence to study drugs.
(4) Patients who show poor treatment response, with worsened symptoms or a ≥ 10% decline in FEV1 compared to baseline at any 13-week assessment visit.
(5) Patients who enroll in other clinical studies.
(6) Patients who voluntarily request to withdraw from the intervention.

Complete loss to follow-up refers to patients with objective inability to follow up due to severe physical impairment, geographical relocation, or confirmed cognitive decline, resulting in the inability to conduct any outcome assessments. These patients will be withdrawn from the study, and their data will be handled according to the ITT principle with missing data addressed using mixed-effects models for repeated measures and multiple imputation in sensitivity analyses.

### 2.4 Sample size

The frequency of acute exacerbations is the primary outcome. According to the data in previous studies, the annual frequency of acute exacerbations in the control group was 1.08 episodes (27), and the experimental group is expected to have a 40% reduction (28), i.e., 0.648 episodes. The test level (*α*) and power (1 − *β*) of the hypothesis test were set at 0.05 and 0.9, and the sample size ratio of the experimental group and control group was 1:1. The estimated sample size for each group was 359 participants using PASS software. After accounting for a 15% dropout rate, the required sample size per group was adjusted to 423 participants. Due to the centralized blocked randomization with block sizes of 4, the sample size per group was adjusted to 424 participants. A total of 848 subjects will be enrolled in this trial.

### 2.5 Randomization, allocation, and blinding

#### 2.5.1 Randomization and allocation

Randomization was performed using the block randomization method supervised by statisticians from the Good Clinical Practice (GCP) Center at the First Affiliated Hospital of Henan University of Chinese Medicine. The drug codes and randomization sequences were generated using SAS software (Version 9.4) and securely archived at the First Affiliated Hospital of Henan University of Chinese Medicine. Before the trial commenced, pre-prepared blinded drugs and corresponding emergency unblinding envelopes were allocated to each center by consecutive number segments. Drug dispensing at each center followed the ascending order of available numbers, with eligible participants receiving medications according to their enrollment order.

#### 2.5.2 Blinding

The blinding procedure was supervised by statisticians from the GCP Center of the First Affiliated Hospital of Henan University of Chinese Medicine and executed by personnel not directly engaged in the trial. Initially, blinded personnel validated the uniformity of study drugs’ appearance and packaging across all groups. Thereafter, drug labels corresponding to assigned drug codes were attached to the outer packaging of each group’s medications. The entire blinding process was documented, with records safely archived for traceability.

Emergency unblinding envelopes containing group allocation information matching the drug codes were sealed and dispatched to each center alongside prepackaged blinded drugs. In emergency situations, if investigators deemed it necessary to identify the administered drug for adverse event management, unblinding could be performed. The unblinding investigator was required to document the rationale, date, and sign on the emergency envelope at the time of opening. Notably, any investigator involved in the emergency unblinding procedure will be excluded from all subsequent assessments and data collection for the unblinded patient to avoid introducing bias. These patients will be followed up and evaluated by other blinded investigators who remain unaware of the group allocation.

### 2.6 Interventions

Both the experimental group and control group will receive standard treatment as outlined in the “GOLD 2023” (24). Standard treatments permitted in this trial include dual bronchodilators (long-acting muscarinic antagonists [LAMAs] and long-acting beta-2 agonists [LABAs]), triple therapy (inhaled corticosteroids [ICS] combined with LAMA/LABA) for patients with ≥2 moderate exacerbations or ≥1 leading to hospitalization within the past year, and short-acting bronchodilators (e.g., salbutamol) for rescue use. Additionally, the experimental group will be given BFYS granules, while the control group will receive BFYS placebos as an adjunct to standard treatment. The treatment regimen will consist of administering two packages of granules twice daily for 52 weeks. Prohibited treatments include other Chinese herbal preparations with similar formulations or therapeutic effects to BFYS granules, as well as other pharmacological and non-pharmacological treatments that have therapeutic effects on COPD.

BFYS granules and placebos are prepared by Jiangyin Tianjiang Pharmaceutical Co., Ltd. The ingredients of placebos include 5% by weight of the original herbs used in BFYS granules and 95% pure dextrin. This formulation is designed to match BFYS granules in appearance, odor, and taste, which is critical for maintaining double-blinding in TCM trials where sensory differences could unblind participants. Notably, the 5% herbal component is substantially lower than the therapeutic dose of BFYS granules. This proportion is too low to produce meaningful pharmacological effects that could impact the trial’s outcomes. Additionally, the primary and key secondary outcomes are objective, quantifiable metrics, reducing the potential for bias from any minimal, subtherapeutic effects. To ensure the consistency in taste and smell between BFYS granules and the placebo, five professionals not involved in this trial conducted a double-blind sensory evaluation. They scored both using a 1–5 points scale in terms of bitterness, herbal flavor intensity, smell, etc. The results showed that there was no statistical difference in the scores between the two groups (*p* > 0.05), confirming their sensory equivalence. In addition, the appearance, weight, and color of the placebos are the same as those of BFYS granules. All drugs meet national quality standards. The drug components of BFYS granules are shown in Table 1.

**Table 1:** Components of BFYS granules.

Chinese name	Latin name	Parts of the substances	Weighting (%)
Ren Shen	*Radix Ginseng*	Root	6.82
Zhi Huang Qi	*Astragali Radix*	Root	11.36
Jiu Yu Rou	*Fructus Corni*	Sarcocarp	9.09
Gou Qi Zi	*Lycii Fructus*	Fruit	9.09
Cu Wu Wei Zi	*Schisandrae Chinensis Fructus*	Fruit	6.82
Zhi Yin Yang Huo	*Epimedium brevicornu Maxim*	Whole herb	6.82
Zhe Bei Mu	*Fritillaria thunbergii Miq*	Bulb	6.82
Chi Shao	*Paeoniae Radix Rubra*	Root	6.82
Di Long	*Pheretima*	Drying body	9.09
Chao Zi Su Zi	*Perilla frutescens*	Seed	6.82
Ai Di Cha	*Japanese Ardisia Herb*	Dried whole grass	11.36
Chen Pi	*Citrus reticulata Blanco*	Peel of fruit	9.09

### 2.7 Outcomes

#### 2.7.1 Primary outcome

The primary outcome is the frequency of AECOPD after the interventions. AECOPD is defined as an acute exacerbation process characterized by dyspnea and/or increased coughing and expectoration (11). The frequency will be recorded every 13 weeks, and the total number over the 52-week treatment period will be calculated. A lower number in a specified period indicates better efficacy.

To ensure complete documentation, participants will record daily respiratory symptoms in the specialized subject diary. For AECOPD-related symptoms, details including onset time and specific symptoms must be documented. Diaries will be submitted weekly and reviewed by researchers every week. Non-hospitalized exacerbations will be verified at the 13-week follow-up using the diary, patient reports, and electronic medical records. For hospitalizations at study sites, the team will retrieve medical records within 48 h using the patient-signed authorization. For hospitalizations at external facilities, patients must notify the team within 72 h and provide complete hospitalization records. The total number of exacerbations will be summarized, confirmed, and entered into the Case Report Form (CRF) at each 13-week visit.

#### 2.7.2 Secondary outcomes

Secondary outcomes include the following indicators:

(1) Number of AECOPD episodes leading to hospitalization: Hospitalizations due to AECOPD should be promptly recorded in the study diary. The frequency should be recorded every 13 weeks during the study period.
(2) Mortality: At the end of the study, the mortality of the two groups will be measured, including the AECOPD-related mortality and all-cause mortality.
(3) Lung function: Tests will include forced vital capacity (FVC), forced expiratory volume in 1 second (FEV1), FEV1% predicted, and FEV1/FVC. Measurements will be conducted at baseline and every 13 weeks during the study period.
(4) Clinical symptoms and signs scores: Assessments will cover cough, expectoration, wheezing, chest tightness, fatigue, cyanosis, etc. Each item will be scored on a 0–3 scale. Higher scores indicate worse disease status. Evaluations will be performed at baseline and every 13 weeks after the start of the trial.
(5) Six-minute walking distance (6MWD): The total distance walked by patients on a flat surface within 6 min reflects exercise capacity. A greater distance indicates better exercise capacity (29). 6MWD will be measured at baseline and every 13 weeks during the study period.
(6) Quality of life: The COPD assessment test (CAT) and 36-item short-form health survey (SF-36) are used to assess the quality of life of patients. A higher SF-36 score indicates better quality of life, while a higher CAT score indicates worse quality of life (30, 31). These questionnaires will be administered at baseline and every 13 weeks after trial initiation.

#### 2.7.3 Safety outcomes

Safety outcomes include blood routine, urine routine, liver function, kidney function, and electrocardiogram (ECG), which are tested once every 13 weeks. Adverse events will be monitored and recorded continuously throughout the study.

#### 2.7.4 Health economics evaluation

Costs refer to the direct and indirect costs incurred by patients for diagnosis and treatment, including the costs of hospitalization, outpatient, vaccine prevention, health care, and psychological treatment. Effectiveness evaluation indicators include the number of AECOPD episodes and the number of AECOPD episodes leading to hospitalization.

The cost-effectiveness analysis method will be adopted to assess the cost saved per unit of effectiveness improvement achieved by BFYS granules. Additionally, we will construct a micro-simulation model of COPD cost-effectiveness evaluation based on Monte Carlo simulation, and conduct basic analysis and sensitivity analysis of the model.

The schedule for enrollment, interventions, and outcomes is presented in Table 2.

**Table 2:** The schedule of enrollment, interventions, and outcomes.

Time point	Study period					
	Enrollment	Baseline	Post-allocation			
	2 weeks	Day 0	13 weeks	26 weeks	39 weeks	52 weeks
Enrollment						
General information	√					
Medical history	√					
Informed consent	√					
Eligibility screen	√					
Randomization		√				
Interventions:						
BFYS formula	6.5 g/pack, 2 pack, twice a day					
BFYS placebo	6.5 g/pack, 2 pack, twice a day					
Outcomes						
Number of AECOPD			√	√	√	√
Number of AECOPD leading to hospitalization			√	√	√	√
Mortality rate			√	√	√	√
Lung function		√	√	√	√	√
Clinical symptoms and signs		√	√	√	√	√
6MWT		√	√	√	√	√
CAT, SF-36		√	√	√	√	√
Blood routine		√	√	√	√	√
Urine routine		√	√	√	√	√
Liver function		√	√	√	√	√
Kidney function		√	√	√	√	√
ECG		√	√	√	√	√
Chest CT		√				
Adverse events			√	√	√	√
Healthcare costs		√	√	√	√	√

AECOPD, acute exacerbations of chronic obstructive pulmonary disease; BFYS, Bufei Yishen; CAT, COPD assessment test; ECG, electrocardiogram; SF-36, 36-item short-form health survey; 6MWT, 6-min walking test.

### 2.8 Data management and monitoring

#### 2.8.1 Data management

We will use the CRF for data collection, which includes general information, laboratory examinations, health questionnaires, health costs, and so on. Researchers will ensure that the CRFs are filled out accurately and completely. Any incorrect records will be re-filled with the correct information, along with the researcher’s signature and date of the day.

Epidata software (Version 3.1) will be used to establish the database. To ensure the timeliness and accuracy of the data, a double-entry approach will be adopted during data entry. During the data preprocessing phase, computerized audits will be carried out to detect missing values, logical errors, and inconsistencies. Based on the audit findings, original CRFs will be reviewed, or researchers will be contacted for verification, so as to guarantee the traceability of the original data.

After the study concludes, all centers will submit the CRFs to the Respiratory Department of the First Affiliated Hospital of Henan University of Chinese Medicine. Research medical records will be managed, stored, and destroyed in accordance with relevant policies. The research data will be locked in cabinets containing participants’ information to ensure their privacy.

#### 2.8.2 Quality control

In order to ensure the quality of this trial, we will establish a quality control committee and a data monitoring committee (DMC). DMC comprises 3 independent experts (1 statistician, 1 pulmonologist, and 1 TCM specialist) with no affiliations to the sponsors or research centers. The committee conducts monthly evaluations on the following aspects: protocol adherence (consistency of interventions, compliance with inclusion and exclusion criteria), data accuracy (verification of 10% of CRFs against original documents), and safety profile (analysis of adverse events). Researchers will comply with the guidelines of good clinical practice (GCP). Quality control experts will conduct monthly quality control assessments to ensure the quality of the study. Additionally, all researchers will receive standardized training on the study protocol. Last but not least, enhancing participants’ compliance is another important measure for quality control. Strategies to improve compliance include monthly telephone follow-ups with participants, quarterly home visits for participants, provision of health education materials, and offering appropriate subsidies, etc.

Additionally, an interim analysis will be conducted by the DMC when 50% of follow-up data are accumulated, assessing efficacy, safety, and futility using the O’Brien-Fleming alpha spending function to control type I error. The DMC will recommend continuation, early termination, or modification based on results. Blinding effectiveness will be evaluated post-trial via questionnaires for investigators and participants to guess group allocation. Correct guess proportions will be compared with 50% (random chance) using the chi-square test; non-significant deviation indicates successful blinding.

### 2.9 Statistical analysis

SPSS software (Version 25.0) will be used for statistical analysis in this study. The study data will be analyzed according to the ITT principle, where all randomized participants will be included in the analysis according to their original group assignment, regardless of protocol deviations or different follow-up durations. For participants with varying follow-up durations, mixed-effects models for repeated measures will incorporate time as a covariate to utilize available data. Missing data will be handled using mixed-effects models for repeated measures as the primary method, with multiple imputation conducted as a sensitivity analysis. Continuous variables will be expressed as either mean ± standard deviation (assuming normal distribution) or median (interquartile range) (non-normal distribution). For the primary outcome, Poisson regression or negative binomial regression will be used based on overdispersion tests (assuming Poisson or negative binomial distribution). For secondary outcomes: count data will use the same methods as the primary outcome; binary data will use logistic regression (assuming binomial distribution); continuous data will use repeated-measures analysis of variance (for normal distribution) or Friedman test (for non-normal distribution). Mauchly’s test will first be applied to assess sphericity for repeated measures. If the assumption is violated (*p* < 0.05), the Greenhouse–Geisser correction will be implemented. Pairwise comparisons within groups will use the Bonferroni method, between groups the LSD-*t* test (equal variances) or Tamhane’s T2 method (unequal variances). Categorical variables will be presented as frequency (%) and compared using the chi-square test (large samples) or Fisher’s exact test (small samples with <5 expected counts). Outliers will be identified using box plots (values beyond 1.5 × interquartile range), verified for data accuracy, and retained in the primary analysis; sensitivity analyses excluding outliers will be conducted. For all statistical tests, both *p*-values and 95% confidence intervals will be reported. A hierarchical testing strategy will be used for multiple comparisons: the primary outcome will be tested first; if significant, secondary outcomes will be tested with Bonferroni correction to control type I error, aligning with the outcome hierarchy. A *p*-value <0.05 is considered statistically significant.

## 3 Discussion

AECOPD is an important cause of the progression and deterioration of COPD. The occurrence of frequent acute exacerbations worsens patients’ quality of life, accelerates the decline in lung function, and even increases the risk of death (9). Frequent hospitalizations also impose a heavy financial burden on patients and society (32). Therefore, reducing the frequency of acute exacerbations is a key goal in COPD management. However, there are few therapeutic regimens and related studies aimed at reducing the frequency of AECOPD. As a result, many patients turn to TCM for solutions (33–36).

BFYS granules, a traditional Chinese medicine (TCM) formulation, have demonstrated multiple therapeutic effects in previous studies, including improving airway epithelial cell aging, protecting the airway epithelial barrier, inhibiting the inflammatory response, and regulating immunity (19–21). This study aims to evaluate the potential benefits of BFYS granules for patients with the frequent exacerbator phenotype in the stable phase of COPD through a multicenter, randomized, double-blind, placebo-controlled clinical trial. Previous studies on BFYS granules (17, 18) showed efficacy in improving COPD symptoms and lung function but did not target the frequent exacerbator phenotype. This trial will fill this gap by focusing exclusively on this population. Furthermore, this study will evaluate the health economic value of BFYS granules, aiming to clarify its economic utility in this population through cost-effectiveness analysis.

If the trial results are positive, BFYS granules may provide a new treatment option, offering broader therapeutic opportunities for stable COPD patients with the frequent exacerbator phenotype. We anticipate that BFYS granules will reduce the frequency of AECOPD and yield improvements in AECOPD-related hospitalizations, lung function tests, exercise capacity, clinical symptoms and signs, quality of life, and cost-effectiveness outcomes.

A key strength of this trial is its rigorous multi-center, randomized, double-blind, placebo-controlled design, which reduces bias and strengthens the reliability of results. Additionally, it also adopts comprehensive assessment outcomes, offering a holistic evaluation of BFYS granules. However, this study has some challenges. First, the intervention period of this trial is 52 weeks, and long-term medication and follow-up procedures may affect patient compliance. Another challenge in this study is the collection of healthcare costs, which requires collecting direct and indirect costs through multiple channels such as patients’ medical records, medical institutions’ billing systems, and patients’ self-reports. In the further, we will explore the efficacy of BFYS granules in other COPD phenotypes and identify its core active components via advanced omics technologies to clarify the underlying mechanisms. Furthermore, we will conduct a long-term follow-up studies to assess sustained effects of BFYS granules.

## Trial status

This study was approved by the Ethics Committee of the First Affiliated Hospital of Henan University of Chinese Medicine (No. 2024HL-043-01) on February 4, 2024. It was registered on ClinicalTrials.gov (NCT06326658) on March 22, 2024. Subject recruitment started on July 1, 2024 and is expected to end on December 31, 2025. As of August 17, 2025, 582 subjects have been enrolled. Data collection is anticipated to be completed by December 31, 2026, with data analysis scheduled to begin on March 1, 2027.
